# Extraction and Functional Properties of Crude Prolamin from Amaranth

**DOI:** 10.3390/foods14223926

**Published:** 2025-11-17

**Authors:** Yujun Dong, Xiaojun Hu, Yajuan Wang, Li He

**Affiliations:** 1 School of Materials and Chemical Engineering, Ningbo University of Technology, Ningbo 315211, China; 6250606060@stu.jiangnan.edu.cn (Y.D.); huxiaojun0316@163.com (X.H.); 2 School of Chemical and Material Engineering, Jiangnan University, Wuxi 214122, China; 3 College of Food Science, Sichuan Agricultural University, Ya’an 625014, China; helifood@163.com; 4 Zhejiang Institute of Tianjin University, Ningbo 315201, China

**Keywords:** response surface methodology, structure, functionality

## Abstract

The objective of this study was to evaluate the structural, functional and morphological characterizations of ultrasonic-assisted extraction of crude prolamin from amaranth grain (*Amaranthus hypochondriacus* L.). A Box–Behnken design of response surface methodology (RSM) was employed to optimize the extraction parameters. The optimal extraction parameters included a solid/solvent ratio of 1:9 (g:mL), with 50% ethanol solution at 75 °C. Regarding the physicochemical characteristics, amaranth crude prolamin (ACP) possessed more orderly secondary structures (the proportions of α-helix and β-sheet were 43.65% and 25.52%, respectively), which is favorable for improving the object-holding capacity, such as higher oil- and water-holding capacity. The higher surface hydrophobicity of ACP was beneficial for self-assembly into microspheres at high ethanol concentrations. In general, ACP had a wider molecular weight distribution, higher zeta-potential and better emulsifying capacity (3.91 g VE/g prolamin). Thus, these results provide useful insight into the applications of amaranth prolamin.

## 1. Introduction

Amaranth, also known as “thousand grain sorghum”, is an ancestral annual crop, which originated in Central and South America and has the advantages of rapid growth, adaptability to harsh conditions and heat resistance [1]. Compared with traditional grains such as maize [2] and sorghum [3], amaranth has more protein content and better amino acid composition balance, so it is a familiar and high-quality functional food resource [4]. Furthermore, amaranth is non-toxic to individuals with celiac disease, and it represents a more sustainable alternative to animal protein sources [5]. Current research on amaranth grain is predominantly concentrated on the extraction and utilization of its starch and hydrosoluble protein isolates, as well as the development of amaranth-based functional foods [6]. However, there is a notable scarcity of studies dedicated to the prolamin in amaranth grain.

Amaranth protein is mainly composed of albumins, globulins, prolamins and glutelins. These fractions account for different percentages of the total protein, with albumins being the highest (about 65%), followed by globulins (about 17%), prolamin (about 11%) and glutelin (about 7%) [7]. Prolamin is one of the most widely present proteins in cereals, including the storage proteins found in grain seeds, some glycoproteins in the cell walls of plants and a great number of low-molecular-mass sulfur-rich proteins in cereal seeds [8]. Currently, methods for extracting protein mainly include organic solvent extraction, physical extraction and biological extraction [9]. Ultrasonic-assisted extraction is widely used in the extraction of plant proteins, such as Moringa oleifera protein [10], alfalfa protein [11] and soy protein isolate [12], because of its advantages of higher yield, shorter processing time, less solvent consumption and minimal environmental impact [13]. Moreover, several recent studies have demonstrated that ultrasound-assisted extraction can significantly enhance the functionality of proteins, including their solubility [12], emulsification capabilities [14] and thermostability [15]. To our knowledge, while ultrasonic-assisted extraction technology has been utilized in the extraction of plant proteins, there is no documented report about ultrasonic-assisted alkali extraction and structure–function relationship of amaranth prolamin.

In this study, response surface methodology (RSM) was used to optimize ultrasound-assisted alkali extraction for the preparation of amaranth prolamin, with the aim to obtain high-purity and strong hydrophobicity protein. The structural and functional properties were systematically assessed by a suite of analytical techniques, including SDS-PAGE, FTIR, hydrophobicity, particle size distribution, SEM and vitamin E-emulsion capacity. The results could inform subsequent studies on the delivery of amaranth prolamin as liposoluble active substance.

## 2. Materials and Methods

### 2.1. Raw Materials and Pre-Treatment

R104 Amaranth seed (*Amaranthus hypochondriacus* L.) was supplied by Shandong Seed Industry Groups Co., Ltd. (Jinan, China). After being milled into fine flour using a high-speed grinder (Wuyi Haina Electric Co., Ltd., Jinhua, China), the grain powder was then passed through an 80-mesh sieve (Jinding Standard Screen Factory, Shaoxing, China). Amaranth powder was defatted with n-hexane (1 g powder with 5 mL hexane). After stirring for 30 min at room temperature, the mixture was centrifuged (9000× *g*, 10 min) with a TG16-WS high-speed centrifuge (Xiangyi Laboratory Instrument Co., Ltd., Xiangtan, China). The precipitates after centrifugation were placed under a fume hood for 12 h to evaporate the residual hexane prior to further processing. Rainbow 245 Spectral Protein marker (11–245 kDa) and Coomassie Blue R 250 were obtained from Shanghai Acmec Biochemical Technology Co., Ltd. (Shanghai, China). Ethylenediaminetetraacetic acid (EDTA), glycine, and 5,5′-dithi-bis-(2-nitrobenzoic acid) (DTNB) were all purchased from Tianjin Kaitong Chemical Reagent Co., Ltd. (Tianjin, China). All other chemicals are of analytical grade, without further purification.

### 2.2. Extraction of Amaranth Crude Prolamin (ACP)

Amaranth prolamin was extracted based on the method of kafirin extraction by Muhiwa [16] with slight modification, taking aqueous ethanol solution with different concentrations containing 0.5% sodium metabisulfite (pH 8.5) as extractants. Briefly, the defatted flour (50 g) was dispersed in the mentioned extractant with different liquid/solid ratios, and the mixed solution was stirred continuously in a 42 kHz ultrasonic reactor, followed by centrifugation at 4500× *g* for 10 min (25 °C). The supernatant was collected, and then an equal volume of pre-chilled distilled water (4 °C) was added. The mixed solution was adjusted to pH 6.0, and then the precipitation was collected by centrifugation (8000× *g*, 10 min). The precipitation was freeze-dried for 24 h with Scientz-10N/A freeze-dryer (Ningbo Xinzhi Biotechnology Co., Ltd., Ningbo, China). The protein content (N × 6.25, N means the percentage of nitrogen in the protein sample) was measured using the Dumas total combustion method, i.e., AACC method 46-30.01. The lyophilized samples were stored in a cool and dry place for further analysis.

### 2.3. Structural Characterization

#### 2.3.1. Sodium Dodecyl Sulfate-Polyacrylamide Gel Electrophoresis

The sodium dodecyl sulfate-polyacrylamide gel electrophoresis (SDS-PAGE) pattern of ACP was performed according to the method as described by Ortega with some modifications [17]. Briefly, the sample was dissolved in Laemmli buffer (250 mM Tris-HCl buffer) containing 0.5% bromophenol blue, 70% ethanol, 10% SDS, 50% glycerol and 2.5% β-mercaptoethanol (5 mg/mL). The solution was heated in a bath of boiling water for 10 min to ensure complete denaturation of the sample. Aliquot (10 L) of sample was loaded onto the precast gradient gel (4% stacking gel and 12% separating gel). Electrophoresis was run in a Mini-protein Tetra system (PROTEAN II electrophoresis apparatus, Bio-Rad, Hercules, CA, USA). Electrophoresis was performed at a voltage of 100 V until the indicator dye reached the bottom of the gel. After separation, the gels were stained with Coomassie Blue R250.

#### 2.3.2. FTIR and Protein Secondary Structure

The KBr tableting method was employed to determine the infrared spectrum of the sample. FTIR spectra were recorded using Nicolet 6700 FT-IR spectrometer (Thermo Nicolet Co., Madison, WI, USA) in the wave number range 400~4000 cm^−1^ over 64 scans at room temperature. The proportion of ACP’s secondary structures was measured from the amide I region (1700–1600 cm^−1^) according to the method described by Chen et al. [18]. The data were quantified using Peakfit 4.12 (Systat Software, San Jose, CA, USA).

#### 2.3.3. Sulfhydryl Group Content

The sulfhydryl content (SH) was measured following the method of Yang et al. with minor modifications [15]. Specifically, about 15 mg of ACP powder was dissolved in 3 mL 70% (*v*/*v*) ethanol aqueous solution, then mixed with Tris-Gly buffer (15 mL, pH 8.0, Gly 0.09 M, Tris 0.086 M and EDTA4 mM), followed by adding 50 μL of Ellman’s reagent (12 mg DTNB, 3 mL Tris-Gly buffer solution). The mixed solution was incubated for 1 h at 25 °C and then centrifuged (8000× *g*, 10 min). The absorbance was recorded using Cary 60 UV-Visible spectrophotometer (Agilent Technology Company, Santa Clara, CA, USA) at 412 nm, and the Tris-Gly buffer was used as a blank. The free SH was calculated as Formula (1).

(1) $$SH(\mu mol/g)=\frac{73.53\times A_{412}\times D}{C}$$

where 73.53 is derived from 10^6^/(1.36 × 10^4^); 1.36 × 10^4^ is the molar extinction coefficient of Ellman’s content; A_412_ indicates the sample’s absorbance at 412 nm; D is the dilution factor of the solution; and *c* represents the sample concentration in mg/mL.

#### 2.3.4. Water Contact Angle (WCA)

The WCA of prolamin was measured using the OCA 15 EC (Dataphysics Instruments Gmbh, Stuttgart, Germany) following the method described by Shi et al. with some modifications [19]. The sample powder was pressed into disks with a diameter of 13 mm and a thickness of 2 mm. The disks were fixed on a glass slide and a drop of deionized water (4 μL) was lightly dropped onto the surface of the disk using a high-precision injector. The images were captured immediately at ten different positions, and the contact angles were analyzed using the SCA20 software (Dataphysics Instruments Gmbh, Stuttgart, Germany).

#### 2.3.5. Particle Size Distribution and ζ-Potential

The method was referred to by Mao et al. with minor modifications [20]. The ACP sample was dissolved into 60% (*v*/*v*) ethanol with a concentration of 1 mg/mL. The dispersive refractive index of the sample was 1.450, and that of the continuous phase was 1.331. Particle size and ζ-potential of the solution at an equilibrium time of 120 s were measured using a laser diffraction particle size distribution Zeta-sizer analyzer (NanoBrook Omni, Brookhaven Instruments, Suffolk, NY, USA). The test was repeated three times at 25 °C.

### 2.4. Functional Properties of ACP

#### 2.4.1. Water- and Oil-Holding Capacity

Water- and oil-holding capacity were acquired using the method described by Shen et al. with some modifications [21]. Briefly, 0.1 g of the ACP sample was thoroughly mixed with distilled water (3 mL) or soybean oil (5 mL) in the centrifuge tube. For water-holding capacity (WHC), the tube was heated at 60 °C for 30 min, followed by centrifugation (8000× *g*, 10 min at 25 °C). For oil-holding capacity (OHC), the centrifugation conditions were 3000× *g* and 10 min at 25 °C. WHC and OHC were calculated based on the weight-gain method.

#### 2.4.2. Emulsifying Capacity for Vitamin E

The emulsifying capacity (EC) denotes the maximum amount of oil that is emulsified under high speed shear conditions. EC of ACP was evaluated using the amount of vitamin E (VE) that can be emulsified by each gram of prolamin. EC and emulsion stability (ES) of ACP for vitamin E were referred to by Mao et al. with some modifications [20]. The VE concentration of emulsion was determined using the HPLC method as described in our previous study [22]. The ACP sample was dispersed in 70% (*v*/*v*) ethanol solution to obtain a concentration of 2 mg/mL; VE was added into the suspension with a ratio of 5:1 (*w*/*w*, 5 g VE and 1 g prolamin) and then homogenized for 3 min using a High-speed Dispersion Homogenizer (HR-500D, Huxi Instrument Co., Ltd., Shanghai, China) with 10,000 rpm at 25 °C. The emulsion (2 mL) was transferred into a 10 mL volumetric flask at 0, 24, 72 and 120 h, into which 10 mg of protease was added. The liquid was fully mixed and left for 30 min at 30 °C, and then 100 μL of the mixture was diluted to 10 mL using ethanol. The solution was analyzed using Agilent 1260 Series HPLC (Agilent Technology Company, Santa Clara, CA, USA) system consisting of a G1312 pump and an automatic injector with a 20 μL loop. Methanol was used as the mobile phase, with a flow rate and detection wavelength of 1.0 mL/min and 285 nm, respectively.

### 2.5. Morphological Properties

The morphology of the sample was carried out using scanning electron microscopy (Hitachi S4800, Hitachi, Ltd., Tokyo, Japan). The lyophilized sample was dissolved in 60%, 70% and 80% (*v*/*v*) ethanol solution, and then were dropped on single-crystal silicon-wafer surfaces and freeze-dried at −48 °C. Prior to obtaining SEM micrographs, the samples were coated with a layer of gold using an ion sputter-meter. Observation was conducted under vacuum conditions at an accelerating voltage of 10 kV.

### 2.6. Statistical Analyses

All the experiments were performed in triplicate and data were expressed as mean ± standard deviation. An analysis of variance (ANOVA) was performed to study model fit using SPSS version 26.0 software (IBM Co., Chicago, IL, USA).

## 3. Results and Discussion

### 3.1. Optimization of ACP Extraction

Based on the extreme point analysis (Figure S1, Tables S1 and S2) using Design-Expert 10.0 (Stat-Ease, Minneapolis, MN, USA), the optimum extraction parameters for amaranth prolamin were obtained as follows: extractant-to-amaranth ratio, 9.42 mL/g; EtOH concentration, 49.93%; and temperature, 74.65 °C. To validate the effectiveness of the model, experiments were conducted under the following conditions: extractant-to-amaranth ratio, 9 mL/g; EtOH concentration, 50%; and temperature, 75 °C. The ACP yield was 9.65 ± 0.04%, closely approximating the predicted value by the model (9.81%). All subsequent ACP used for further experiments were prepared in these optimized conditions; the protein purity of the sample was 90.41%. The content of prolamin varies significantly for various cereals. Overall, the extraction yield of amaranth prolamin is higher than that of other cereals, such as oat, rye, and sorghum [23]. Furthermore, both the acoustic cavitation effect in the ultrasonic field and the presence of reducing agents can contribute to the solubility of the proteins, thereby enhancing the extraction yield.

### 3.2. SDS-PAGE

**Figure 1:**
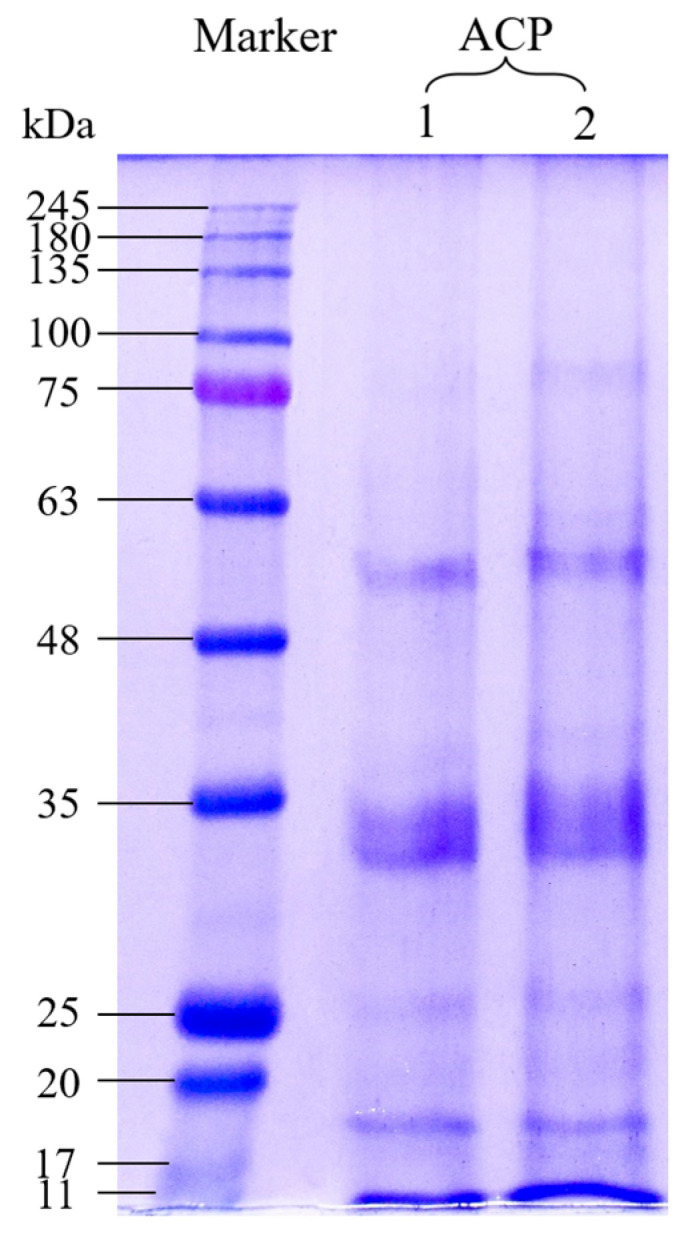
SDS-PAGE profile of ACP (1 and 2 are parallel experiments).

SDS-PAGE is commonly used to provide information about the molecular weight (M_w_) distribution of protein. Figure 1 displays the profiles of the ACP sample under reducing conditions with a specific molecular weight marker. As shown, the M_w_ of ACP was mainly distributed between 17 and 63 kDa, revealing the presence of acidic and basic subunits of protein with M_w_ of 32~36 kDa and 19~28 kDa, respectively [24]. Furthermore, the less significant band at 50.2~56.8 kDa was known as the subunit of homohexamer assembled by preglobulin, containing acidic and basic peptide segments linked by disulfide bonds [25]. Leyva-Lopez et al. found five bands between 22 kDa and 52 kDa for amaranth protein [26]; the different band numbers and molecular weight distribution range may be due to different extracting processes and amaranth varieties. Generally, almost all physicochemical properties of proteins, including emulsifying property, gel property, solubility and film formation, are closely related to the molecular weight [27]. Thus, the significant differences in the M_w_ distributions between ACP and other prolamins will result in markedly different functionalities. Meanwhile, the preglobulin structure in the ACP molecule also makes it easier to form amyloid fibers, which need to be further explored.

### 3.3. Secondary Structure

**Figure 2:**
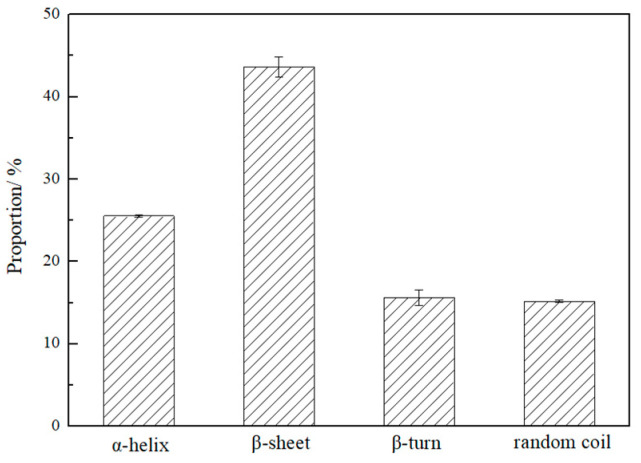
Secondary structure proportion.

The percentage of secondary structure in ACP derived from the amide I band (1700~1600 cm^−1^ from FTIR spectrum) was calculated according to Chen et al., where 1600~1640 cm^−1^ belongs to the β-sheet, 1641~1650 cm^−1^ corresponding to random coil, 1651~1660 cm^−1^ is for α-helix, 1661~1685 cm^−1^ and 1686~1700 cm^−1^ represent β-turn and reverse parallel β-sheet, respectively [18], and the proportion of secondary structure can be calculated using the areas of these peaks. As shown in Figure 2, the proportion of each secondary structure in descending order is as follows: β-sheet > α-helix > β-turn > random coil, indicating that ACP is rich in β-sheet structures. Studies indicate that the content of the β-sheet may be decreased in an ultrasonic field [28]. Moreover, the β-sheet structure in protein is formed by regular hydrogen bonding between an amino group (NH_2_) and carbonyl group (C=O) on the main chains of adjacent peptide chains, forming a jagged lamellar structure [23]. For prolamin, a large number of β-sheet conformations are readily triggered to reverse hydrophobicity, resulting in the formation of head-to-tail connected bands, and then continue to curl and eventually self-assemble into nanoparticles [29]. Compared with zein (24.7% β-sheet [23]), ACP contains a higher proportion of β-sheet; therefore, ACP is theoretically more prone to self-assembly into micro/nanoparticles, which requires further verification.

### 3.4. Sulfhydryl Content and WCA Analysis

The free sulfhydryl groups in protein molecules have a significant effect on stability and functionality, as presented in Table 1. As shown in the table, the -SH_free_ of the ACP was remarkable compared to those of white quinoa (*Chenopodium quinoa Wild.*) protein isolate (11.30 μmol/g, [21]) and was higher than zein. The acoustic cavitation effect can change the spatial structure of protein, thereby exposing the internal sulfhydryl groups; furthermore, the partial disulfide bonds can be broken, which both increase the content of -SH_free_. Meanwhile, free sulfhydryl groups can scavenge radicals, maintaining normal cellular metabolism and protect cell membrane integrity [30]. Therefore, it can be inferred that ACP has better oxidation resistance, which needs to be further verified.

WCA is the angle between the solid–liquid interface and the gas–liquid interface at the junction of the solid, liquid and gas phases. The surface of the solid is hydrophilic if the angle is less than 90°, which means that the liquid can wet the solid easily. The WCA data are shown in Table 1. To be used for food preservation and pharmaceutical slow-release agents, the main materials should possess sufficient water repellence. Compared with commercial zein (WCA was 53.91°), ACP has a more pronounced hydrophobicity. The application of ultrasonic wave has a positive effect on the disaggregation of ACP because of the shear force and shock wave, which promotes the breaking and depolymerization of non-covalent bonds, releasing hydrophobic regions previously encapsulated within the prolamin aggregates [31]. Simultaneously, the WCA of ACP and zein is less than 90°, which does not indicate that the sample is hydrophilic. It is well known that the roughness of sample surface can significantly affect the WCA [32]. The surface of the testing sample may not be smooth due to the insufficient pressure.

### 3.5. Particle Size and ζ-Potential Analysis

**Figure 3:**
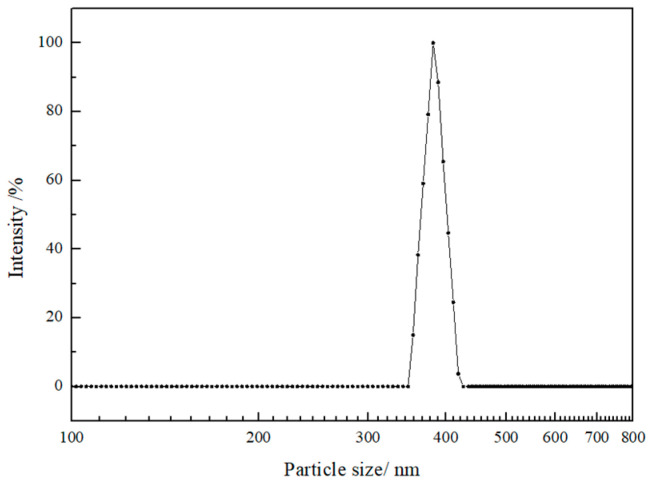
Particle size distribution in 60% ethanol solution of ACP.

The protein particle size and ζ-potential are the most important factors affecting various physicochemical and functional properties, such as solubility, emulsification and dispersibility. Zeta-potential represents the electrostatic repulsion between protein particles, which is related to the surface charge distribution of the particles in the suspension. The higher the potential, the greater the repulsive force between particles, and thus, the more stable for the solution [33]. The particle size of ACP is shown in Figure 3, and ζ-potential is listed in Table 1. It can be found that the average particle size of ACP in 60% (*v*/*v*) ethanol solution was 346.5 nm, the polydispersity index (PDI) was 0.108, and the ζ-potential of ACP sample was 18.92 mV, higher than that of zein in the ethanol solution of the same concentration (6.40 mV), indicating that ACP solution shows a more stable colloidal system. ACP has a relatively large molecular weight (Figure 1) but a small particle size, indicating a more compact spatial structure. The smaller particle size and higher ζ-potential of ACP may be attributed to the utilization of ultrasonic waves, which separate induced charges and expose positively and negatively charged amino acids, causing increased electrostatic repulsion [31]. Similar results were found in Dabbour et al.’s research, in which the authors observed that the cavitation effect of the ultrasound improved the absolute value of ζ-potential [34].

### 3.6. Oil- and Water-Holding Capacity

OHC and WHC indicate the ability of oil and water retention of ACP, which is listed in Table 1. The OHC of protein generally expresses the interaction between protein and lipids, and is a critical attribute in determining the emulsifying properties and flavor retention. On the other hand, WHC is strongly associated with gelation properties and the viscosity and texture of food products. As shown, OHC and WHC of ACP were significantly higher than those of crude prolamin from kidney beans (*Phaseolus vulgaris* L.) (2.02~3.44 g oil/g prolamin, and 0.59~1.25 g water/g prolamin) and common pulse proteins (0.89~1.80 g oil/g prolamin, and 1.2~4.9 g water/g prolamin) [15,35]. Generally speaking, the two characteristics are mainly affected by amino acid composition, structural conformation and hydrophobicity of proteins [21]. Overall, ACP exhibits good oil and water retention properties. This is attributed not only to the amino acid composition in its primary structure, but also to the high proportion of α-helix and β-sheet secondary structures in ACP (as shown in Figure 2), which contributes to the superior object-holding capacity.

### 3.7. Emulsifying Capacity and Storage Stability

**Figure 4:**
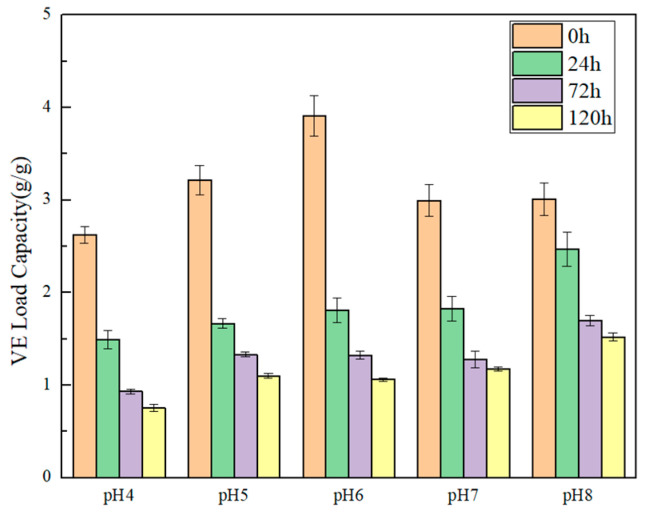
VE-emulsifying capacity and storage stability at different pH of ACP.

Emulsification properties are important in many food applications of protein ingredients. Prolamins possess the potential emulsifying ability mainly because of their amphiphilic properties. The structural and functional characteristics of prolamin can be affected by testing conditions, including the temperature, concentration, ionic strength and pH [36]. The pH dependence of the VE-emulsification capacity and storage stability of ACP emulsion is presented in Figure 4. It was found that when the pH value of the solution is changed, the EC for VE shows significant differences. As shown, the lowest VE-emulsification capacity was observed at pH 4.0. When the pH of the solution was increased to 6 (near the pI of prolamin), ACP demonstrated excellent EC, which was 3.91 g VE/g prolamin. This phenomenon is contrary to the results of Shen et al. for emulsifying properties in quinoa protein [21]. It can be speculated that the emulsification mechanism is different between prolamin and water-soluble protein. The water-soluble protein mainly serves as a molecular emulsifier, which can form a protective layer on the surface of the oil droplet. Hydrophobic amino acid residues can enter the interior of the oil droplet, while the other parts stretch to prevent the hydrophobic interaction of oil droplets. The higher solubility in alkaline condition enhances the emulsification capacity of the water-soluble proteins. However, prolamin exists in the form of rigid nanoparticles in the system, and a large number of nanoparticles are attached to the surface of the oil droplet to maintain the stability of the emulsion. For Pickering emulsifier, there are more prolamin particles at pI, resulting in a higher VE-emulsifying activity index. It is especially interesting to notice that the EC of ACP could compete well with gelatin, which is recognized as a common food emulsifier [37]. The higher EC of ACP could be due to its good hydrophobicity and higher protein purities.

Furthermore, with the extension of storage time, the VE emulsion capacity significantly decreased. Relatively speaking, the emulsion has a better stability at pH 8.0. The higher storage stability at alkaline condition than in neutral solution agrees with the report by Thewissen about gliadins (wheat prolamin) [38]. The good emulsion stability under alkaline conditions was primarily attributed to the hydrophobic group interactions, resulting in the formation of larger aggregates among protein molecules [39], consequently leading to enhanced emulsification stability. VE is easy to saponify when pH exceeds 8; therefore, the EC and storage stability of ACP for VE in a strong alkali environment was not mentioned in this work.

### 3.8. Microscopic Morphology Analysis

**Figure 5:**
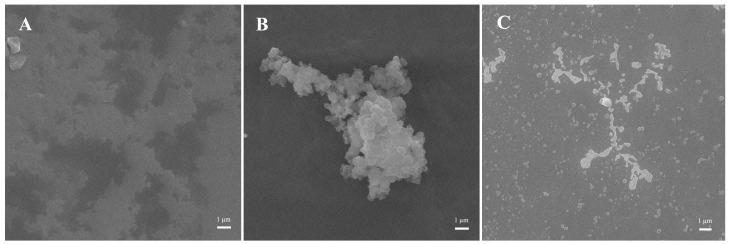
SEM of ACP at different ethanol concentrations ((**A**) 60%; (**B**) 70%; (**C**) 80%).

The microscopic morphology and self-assembly process in different ethanol concentrations were examined to visualize the microstructure and aggregation of ACP (as shown in Figure 5). From Figure 5A, distinct aggregate and membrane structures in 60% ethanol solution can be found. Low concentrations of ethanol exhibit greater polarity and prolamin molecules are more inclined to expose hydrophilic groups to the solvent, leading to the indication of self-assembly into film due to charge repulsion [40]. When the ethanol concentration increases to 70% (*v*/*v*), the electrostatic repulsion prompts the prolamin molecules to shift to a more stable state; thus the prolamin molecules will aggregate with each other to self-assemble into an irregular aggregate structure composed of nanoparticles on top of the particles. As the ethanol concentration increases (80%, as shown in Figure 5C), the hydrophobicity of the solvent increases, prompting the hydrophobic groups in the prolamin to be exposed. In contrast, the volatilize rate of water is lower than that of ethanol, causing the prolamin with hydrophobic groups on the outside to self-assemble into nanoparticles. A similar phenomenon was observed in zein [41]. Additionally, the relatively large proportion of β-sheet structure in ACP (as shown in Figure 2) also makes it prone to forming fibrillar aggregates, as demonstrated in Figure 5C.

## 4. Conclusions

In this study, optimization and functionality of amaranth crude prolamin were obtained using ultrasound-assisted extraction. The optimal parameters by RSM are as follows: solid/solvent ratio was 1:9 (g/mL), ethanol concentration was 50%, and temperature was 75 °C. The structural characterization shows that ACP has a high proportion of α-helix and β-sheet structures. The molecular weight of ACP was mainly distributed between 17 and 63 kDa. The particle size and ζ-potential in 60% ethanol solution were 346.5 nm and 18.92 mV, respectively. For its functional properties, ACP has excellent water- and oil-holding capacity, and can emulsify up to 3.91 g vitamin E per gram prolamin. Moreover, ACP can self-assemble into microspheres and fibrillar aggregates in high ethanol concentrations (80% ethanol aqueous solution). These results are able to provide guidance for the application of amaranth prolamin. In theory, the size and digestibility of emulsion are important factors that may exhibit influence on the bioavailability of the fat-soluble nutrients. More efforts should be placed on studying the simulation of their micro-encapsulation and pharmacokinetics.

## Figures and Tables

**Table 1:** Physicochemical and functional properties of ACP.

Property	ACP	Zein
SH_free_ (μmol/g)	19.51 ± 0.27	6.2 ± 0.19
WCA (°)	77.11 ± 0.21	53.91 ± 0.24
ζ-potential (mV)	18.92 ± 1.08	6.40 ± 0.58
OHC (g/g)	6.34 ± 0.11	8.63 ± 0.20
WHC (g/g)	7.45 ± 0.32	2.21 ± 0.14

## Data Availability

The original contributions presented in this study are included in the article/Supplementary Materials. Further inquiries can be directed to the corresponding author.

## Supplementary Materials

The following supporting information can be downloaded at https://www.mdpi.com/article/10.3390/foods14223926/s1, Figure S1: Three-dimensional interaction and contour plots between single factors; Table S1: Response surface central composite design and yield of prolamin; Table S2: ANOVA table of the response.
