# Supplementary material for: Extraction and Functional Properties of Crude Prolamin from Amaranth
Source: Foods. 2025 Nov 17;14(22):3926. doi: 10.3390/foods14223926 (PMC12651533; doi:10.3390/foods14223926)
Supplement: Supplementary file 1 [file foods-14-03926-s001.zip › foods-3929546-supplementary.pdf]

Results of RSM

RSM methodology was employed to optimize extraction of prolamin from defatted amaranth flour using Box-Behnken design. The yield of every experiment was shown in Table 1, and the results were analyzed using Design Expert 13.0.1.0, which employed to optimize parameters for the extraction process. Results reveal that the experimental prolamin yield varied from 4.481 to 9.721 g/100g amaranth flour. A second-order multivariate regression equation for yield was obtained through multiple linear regression analysis:

$$Y = 9.56 + 0.6614X_1 + 0.0131X_2 + 0.2623X_3 - 0.4352X_1X_2 + 0.824X_1X_3 + 0.3125X_2X_3 - 1.90X_1^2 - 1.93X_2^2 - 0.32X_3^2$$

The quadratic regression equation used a mathematical model to reflect the impact of each independent factor (X_1, X_2, X_3), the quadratic interaction (X_1X_2, X_1X_3, X_2X_3) and the square of the factors (X_1^2, X_2^2, X_3^2) on the prolamin yield. The mathematical relationship obtained by RSM is more comprehensive compared with that of control variable method. The results of the analysis of ANOVA were shown in Table 2. The regression coefficients R^2 and the adjusted R^2_{adj} (Table 2) was 0.9871 and 0.9639, respectively, indicating a reasonable fit of the models to the experimental results. Meanwhile, the coefficient of variation (CV) was 4.57%, indicating the model is credibility reproducible [23]. ANOVA analysis demonstrated that the lack of fit term was not significant (p>0.05), the linear term of EAR, quadratic term of temperature, and all squares had a significant impact on yield, meaning the model was validated. Comparatively speaking, the concentration of ethanol has weaker effect on the yield, which may be due to the better solubility of AP.

The response surface three-dimensional plots and contour plots based on the regression model are shown in Fig. 1. The interaction between the three factors is not a simple linear relationship, and the steepness of the surface plot is positively correlated with the interaction between two independent variables. The steeper the response surface three-dimensional plot and the more elliptical the contour plot, the more significant between factors. As shown in Fig.1, the order of steepness is X_1X_3>X_1X_2> X_2X_3, which stated the interaction of EAR and temperature has the most significant effect on prolamin yield. A similar trend for protein yield was observed by Hadidi et al. [11]. The contour of X_2X_3 was close to circular, indicating the yield of amaranth prolamin was not affected by the interaction between ethanol concentration and temperature (p>0.05). The results were consistent with the ANOVA analysis (Table 2).

Table S1 Response surface central composite design and yield of pure protein

Run	Factors			Response
	X_1: EAR (mL/g)	X_2: EtOH	X_3: Temp (℃)	Yield / %
1	7	0.4	70	4.481±0.207
2	9	0.4	60	6.608±0.109
3	9	0.4	80	6.084±0.149
4	7	0.5	60	6.124±0.261
5	7	0.6	70	5.475±0.182
6	9	0.5	70	9.484±0.077
7	7	0.5	80	5.424±0.084
8	9	0.5	70	9.474±0.161
9	11	0.5	80	8.211±0.328
10	9	0.6	60	5.912±0.117
11	11	0.5	60	5.615±0.181
12	9	0.6	80	6.638±0.117
13	11	0.4	70	6.858±0.211
14	9	0.5	70	9.721±0.069
15	11	0.6	70	6.111±0.105

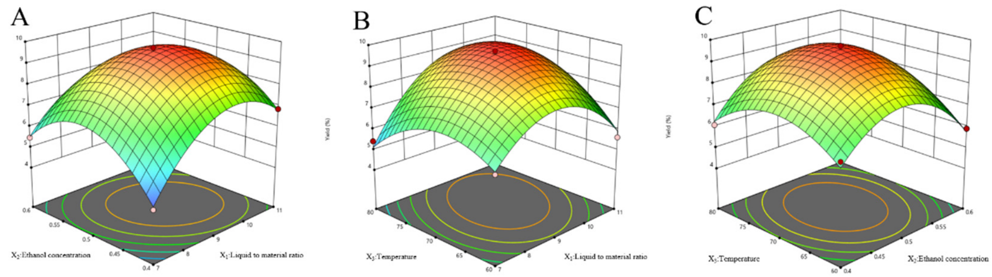

Figure S1 3D interaction and contour plots between single factor

Table S2 ANOVA table of the response

Source	Coefficient estimate	F-value	p-value
Model	37.12	42.59	0.0003
X_1-EAR	3.50	36.13	0.0018
X_2-EtOH	0.0014	0.0142	0.9097
X_3-Temp	0.5502	5.68	0.0629
X_1X_2	0.7578	7.82	0.0381
X_1X_3	2.72	28.04	0.0032
X_2X_3	0.3906	4.03	0.1009
X_1^2	13.30	137.30	<0.0001
X_2^2	13.76	142.12	<0.0001
X_3^2	6.42	66.27	0.0005
Residual	0.4842		
Lack of Fit	0.4451	7.59	0.1186
R^2	0.9871		
Adj-R^2	0.9639		
CV (%)	4.57		
